# Management, clinical outcomes, and its predictors among heart failure patients admitted to tertiary care hospitals in Ethiopia: prospective observational study

**DOI:** 10.1186/s12872-022-03008-7

**Published:** 2023-01-06

**Authors:** Berhanu Beri, Korinan Fanta, Fekede Bekele, Worku Bedada

**Affiliations:** 1 Clinical Pharmacy Course and Research Team, School of Pharmacy, Institute of Health Sciences, Jimma University, P.O.Box: 378, Jimma, Oromia Ethiopia; 2 Institute of Health Sciences, Adama Comprehensive Specialized Medical College, P.O.Box: 84, Adama, Oromia Ethiopia

**Keywords:** Heart failure, All-cause mortality, Outcomes, Ethiopia

## Abstract

**Background:**

Heart failure is a global pandemic, as it affects approximately 64.34 million people worldwide with a $346.17 billion global economic burden. The prevalence of heart failure has increased from 43.4 to 46.5% in the last 10 years in lower and middle-income countries. Most of the studies conducted in Ethiopia were retrospective cross-sectional, with limited study participants, and conducted in a single setting that commonly addresses the prevalence and pattern of heart failure rather than clinical outcome, associated factors, and specific management in different areas. Hence, this study aimed to assess management, clinical outcomes and their predictors among heart failure patients admitted to tertiary care hospitals in Ethiopia.

**Methods:**

A prospective observational study design was conducted on heart failure patients admitted at two tertiary care hospitals in Ethiopia from September 2020 to May 2021. Using semi-structured questionnaires relevant data were collected from patients’ medical records and face-to-face interviewing. Data were analyzed using SPSS version 23.0. A multivariate Cox regression model was performed to identify independent predictors of 90-day all-cause mortality. Variables with P values < 0.05 were considered statistically significant.

**Results:**

Out of 283 patients enrolled in this study, 52.3% were male and the mean (± SD) age was 52.4 ± 17.9 years. The most common medications prescribed during hospitalization and discharge were diuretics (98.9% vs 95.6%), angiotensin I converting enzyme inhibitors/angiotensin II receptor blockers (48.8% vs 67.3%), and beta-blockers (46.6% vs 64.7%), respectively. In the present study, the 90-day all-cause mortality was 10.2%. Hypertension (HR = 3.7, 95% CI 1.2–11.6), cardiogenic shock (HR = 8.7, 95% CI 3.2–20.8), alcohol drinking (HR = 2.8, 95% CI 1.1–7.8), absence of angiotensin I converting enzyme inhibitors/angiotensin II receptor blockers (HR = 0.02, 95% CI 0.0–0.2), and reduced ejection fraction (HR = 1.5, 95% CI 1.1–3.8) were predictors of 90-day all-cause mortality.

**Conclusion:**

High 90-day all-cause mortality was observed among heart failure patients in the present study. In the current study, the majority of heart failure patients were treated with diuretics. Alcohol drinking, hypertension, cardiogenic shock, reduced ejection fraction, and absence of angiotensin I converting enzyme inhibitors/angiotensin II receptor blockers drugs were predictors of poor treatment outcomes for whom restriction of alcohol consumption, early management of hypertension, reduced ejection fraction, cardiogenic shock, and providing angiotensin I converting enzyme inhibitors/angiotensin II receptor blockers drugs for all heart failure patients would be recommended to improve these poor treatment outcomes.

## Introduction

Heart failure (HF) is a complex clinical syndrome that results from any structural or functional impairment of ventricular filling or ejection of blood. [1].

Population-based studies on the incidence and prevalence of HF in developing countries estimated that cardiovascular diseases (CVD) accounted for 7–10% of all medical admissions to African hospitals and HF constitutes 3–7% of these admissions [2]. The prevalence cases of CVD in Ethiopia have increased approximately from 1.4 million in 1990 to 2.84 million in 2017. The disability-adjusted life years (DALYs) and mortality rates were 3549.6 and 182.63, respectively in 2017 [3].

Heart failure is a global pandemic of cardiovascular disease, as it affects approximately 64.34 million people worldwide of who 29.5 million were males and 34.8 million were females, accounting for 9.91 million years lost due to disability. More specifically, the burden of HF has increased by 3.1% in the last 10 years in low-to-middle-income countries (from 43.4 to 46.5% of all worldwide HF cases) [4] and it covers around 44% in Sub-Saharan Africa [5]. Heart failure is responsible for significant health and economic burden. The global economic burden of HF is estimated at 346.17 billion US dollars with 5380 US dollars per HF case as per the American heart association estimation [4].

Many comorbid conditions are associated with an increased propensity for structural heart disease. Hypertensive men and women have a substantially greater risk of developing HF than normotensive men and women. As well, the presence of clinical diabetic Mellitus markedly increases the likelihood of developing HF in patients without structural heart disease, obesity, or insulin resistance as well as increased development of HF [1, 6]**.** The observational data supports the risk of smoking with increased heart failure mortality and rates of hospital admissions due to worsening heart failure compared with never, recent ex-, and longer ex-smokers [7].

Among all causes of HF worldwide in 2017, ischemic heart disease accounted for the highest proportion (26.5%), followed by hypertensive heart disease (26.2%), chronic obstructive pulmonary disease (23.4%), another cardiomyopathy (6.5%), non-rheumatic degenerative mitral valve disease (2.7%), other cardiovascular and circulatory diseases (2.4%), alcoholic cardiomyopathy (2.4%), non-rheumatic calcific aortic valve disease (2.3%), rheumatic heart disease (1.8%), and myocarditis (1.7%) [8]. A recent meta-analysis of studies in sub-Saharan Africa showed that hypertensive heart disease (39%) was the commonest cause of HF in sub-Saharan Africa, followed by cardiomyopathy (21%) and rheumatic heart disease (14%), with ischemic heart disease less frequent (7%) [9].

In the management of HF patients, angiotensin-converting enzyme inhibitors (ACEIs) have been shown to reduce mortality and morbidity in patients with HFrEF. Spironolactone or eplerenone is recommended in all symptomatic patients with HFrEF and ejection fraction ≤ 35%, to reduce mortality and HF hospitalization. In addition, diuretics are recommended to reduce the signs and symptoms of congestion in patients with HFrEF, but their effects on mortality and morbidity have not been studied in randomized controlled trials. Unfortunately, there are no treatment strategies with proven benefits to reduce mortality in patients with HFpEF, but calcium channel blockers and diuretics are used commonly [10–13].

The global study conducted by the international congestive heart failure study (INTER-CHF) among HF patients from low and middle-income countries reported that participants from Southeast Asia had an intermediate rate of mortality at the mean age of 57 years (15%) compared with patients in China, South America, and the Middle East patients who had the lowest rates of death at the mean age of 60 years, 69 years, and 72 years (7.3%, 9.1%, and 9.4%), respectively. However, Patients in India and Africa had the highest mortality at the mean age of 59 years and 56 years (23.3% and 33.6%), respectively [14].

In contrast to developed countries, there are limited studies: most of the studies are retrospective, conducted in a single setting, and a scanty number of participants were recruited on the treatment outcome of HF patients in sub-Saharan African countries, including Ethiopia. The previously studied reports in Ethiopia showed that the prevalence and pattern of heart failure increased. On the other hand, the study previously conducted in Ethiopia did not address short-term clinical outcomes and their predictors among HF patients. Hence, this study finding illustrated 90-day clinical outcomes and their predictors which were used by patients, community, and healthcare providers by creating awareness regarding HF prognostic factors, management, and all-cause mortality among HF patients who were admitted to two tertiary care hospitals in Ethiopia.

## Methods and participants

### Study design and clinical setting

A prospective observational study was conducted at two tertiary hospitals in Ethiopia including Jimma University Medical Center (JUMC) and Ambo University Referral Hospital (AURH). JUMC serves as a referral center for the southwestern part of the country (over 15,000,000 catchment population). JUMC provides general internal medicine, cardiology, and other clinical and diagnostic specialty services. AURH provides service through its medical units similar to JUMC for approximately 64,000 patients each year coming from different areas. The study was conducted from September 14, 2020, to May 30, 2021.

### Study population

**Fig. 1:**
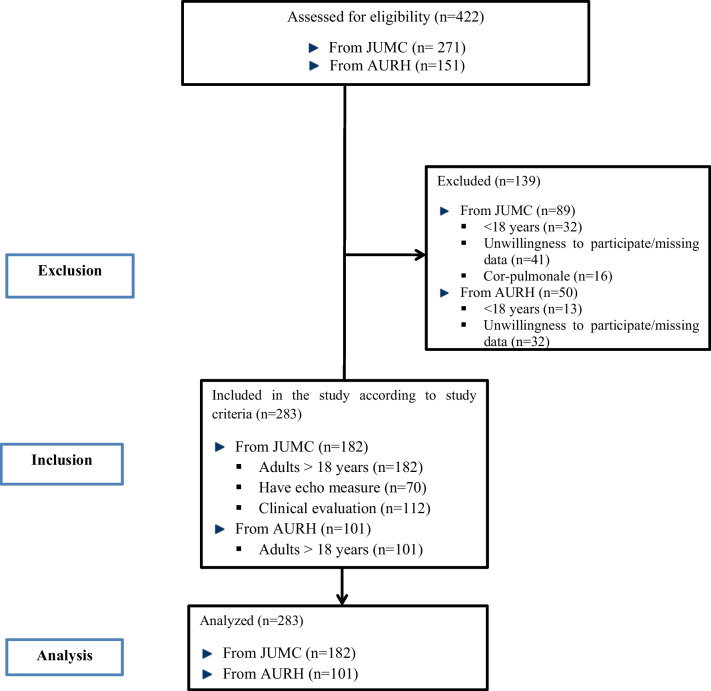
Strobe flow chart of study and sampling population

All consecutive HF patient**s** admitted to JUMC and AURH fulfilling the inclusion criteria were included. Patients were included if they fulfilled the following inclusion criteria: (I) age ≥ 18 years**; (**II**)** both newly diagnosed and known HF patients; and (III) both clinically and echocardiograph-confirmed diagnosis (the suspected heart failure based on signs and symptoms of HF with or without abnormal electrocardiogram (ECG) is settled by echocardiography) of HF patients. Patients who were unwilling to participate in this study and patients with isolated right-sided heart failure (Cor-pulmonale) were excluded from the study (Fig. 1).

### Data collection

Using a pre-tested structured questionnaire, two trained nurses and two pharmacists collected clinical data by interviewing hospitalized patients and extracting relevant data from patients' medical records. The data collection tool includes socio-demographic characteristics, key diagnostics investigations (cardiac troponin, complete blood count, electrocardiography, and echocardiography), medication profiles, and clinical outcomes.

### Study outcomes and validating tools

The primary outcome of the study was 90-day all-cause mortality which was ascertained from the treating physician's death summary note and/or contacting the patient's family via telephone. All clinical endpoints were recorded by following patients on daily basis from admission to discharge or death.

### Statistical analysis

The collected data were checked for completeness, consistency, clarity, and accuracy. Data was entered into Epidata version 3.1 and analyzed using the statistical package for social science (SPSS) version 23. Continuous variables were presented as mean (± SD) for normally distributed variables, and/or median (inter-quartile range) for non-normal distributed variables, and categorical variables are presented as frequency (%). A 90-day all-cause HF mortality rate was compared using Kaplan–Meier and log-rank tests. P value < 0.25 was considered as a cut-off point to select variables on univariate regression for multivariable Cox regression to determine independent predictors of 90-day all-cause mortality. Two-tailed P value < 0.05 was considered statistically significant.

## Results

### Socio-demographic characteristics and behavioral measures

Among 283 HF patients enrolled in the study, 52.3% were male and the mean (± standard deviation) age was 52.4 ± 17.9 years old. Of these, 189 (66.8%) participants were younger than 65 years old, 180 (63.6%) were rural residents, 141 (49.8%) had no formal education, 98 (34.6%) were farmer/labor workers, 192 (67.8%) were non-adherent to salt intake recommendations, and 67 (23.7%) were alcohol drinkers (Table 1).

**Table 1:** Baseline socio-demographic characteristics and behavioral measures of heart failure patients admitted to JUMC and AURH, Ethiopia between September 01, 2020 and March 01, 2021

Demographics	Categories	Frequency (%)
Age (in years)	Mean (± SD)	52.4 ± 17.9
	< 25	23 (8.1)
	25–54	109 (38.5)
	55–64	57 (20.1)
	≥ 65	94 (33.2)
Gender	Female	135 (47.7)
	Male	148 (52.3)
Residence	Rural	180 (63.6)
	Urban	103 (36.4)
Marital status	Single	31 (11.0)
	Married	252 (89.0)
Education level	No formal education	141 (49.8)
	Primary school	30 (10.6)
	Secondary school	47 (16.6)
	Tertiary education	65 (23.0)
Occupational status	Unemployed/retired	83 (29.3)
	Farmer/labor worker	98 (34.6)
	Housewife	54 (19.1)
	Employed (Govt/NGO)	48 (17.0)
Non-adherent to low salt intake	Yes	192 (67.8)
Cigarette smoking	Yes	36 (12.7)
Khat chewing	Yes	80 (28.3)
Alcohol drinking habits	Yes	67 (23.7)

SD-Standard deviation, Govt/NGO-Government or Non-government organization

### Comorbid conditions

**Fig. 2:**
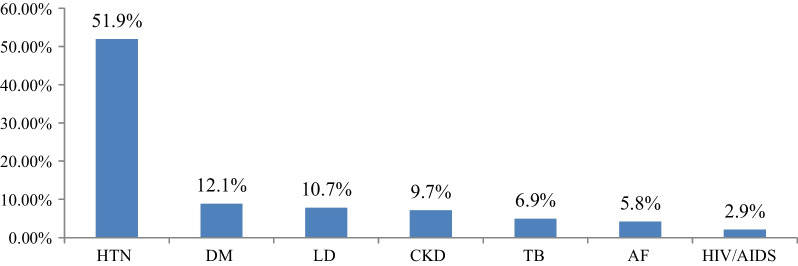
List of common co-morbid diseases that co-exist with HF patients admitted to JUMC and AURH, Ethiopia between September 01, 2020 and March 01, 2021. AF-atrial fibrillation, CKD-chronic kidney disease, DM-diabetic Mellitus, HIV/AIDS-human immune deficiency virus and/or acquired immune deficiency syndrome, HTN-hypertension, LD-liver disease, TB-tuberculosis

Among 283 participants, 206 (72.8%) had documented comorbid conditions. Of these, the most common comorbid conditions in order of decreasing frequency were hypertension 107 (51.9%), diabetic Mellitus 25 (12.1%), and liver disease 22 (10.7%) (Fig. 2).

### Clinical characteristics

**Fig. 3:**
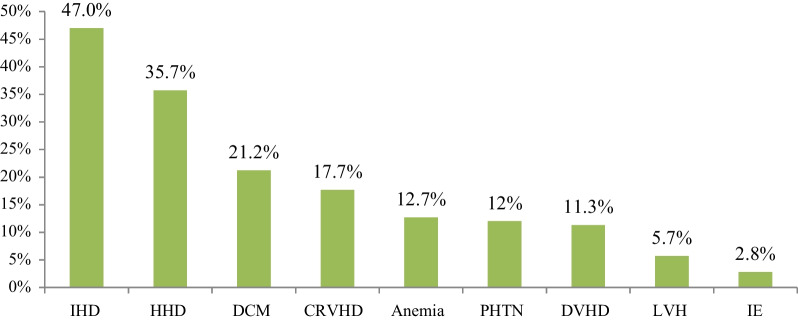
Underlying causes of heart failure patients admitted to JUMC and AURH, Ethiopia between September 01, 2020 and March 01, 2021. CRVHD-chronic valvular heart disease, DCM-dilated cardiomyopathy, DVHD-degenerative valvular heart disease, HHD-hypertensive heart disease, IE-infective endocarditis, IHD-ischemic heart disease, PHTN-pulmonary hypertension, LVH-left ventricular hypertrophy

Among 283 patients included in the current study, 72 (25.4%) and 110 (38.9%) had elevated systolic and diastolic blood pressure, respectively. Two hundred seventy (95.4%) participants had structural heart disease at stage C and 226 (79.9%) patients were functional class IV HF. The leading causes of HF on echocardiography findings were, ischemic heart disease in 133 (47.0%) of the patients followed by hypertensive heart disease in 101 (35.7%), dilated cardiomyopathy 60 (21.2%), and chronic rheumatoid valvular heart disease 50 (17.7%) (Fig. 3). The three most common precipitating factors of HF in the descending order of frequency were community-acquired pneumonia 122 (57.8%), atrial fibrillation 60 (28.4%), and drug discontinuation 49 (23.2%) (Table 2). The reason for drug discontinuation was COVID-19 negative impact 26 (53.1%).

**Table 2:** Clinical characteristics of heart failure patients admitted to JUMC and AURH, Ethiopia between September 01, 2020 and March 01, 2021

Parameters	Frequency (%)
Systolic BP ≥ 130 mmHg	72 (25.4)
Diastolic BP ≥ 80 mmHg	110 (38.9)
*Heart rate (bpm) (n* = *239)*	
< 60 ≥ 100	12 (5.0) 124 (51.9)
*Patient type*	
Newly diagnosed HF Known HF	127 (44.9) 156 (55.1)
*Duration of HF (in years) (n* = *156)*	
≤ 5 > 5	120 (76.9) 36 (23.1)
*Stage of HF*	
B C D	3 (1.1) 270 (95.4) 10 (3.5)
*NYHA class*	
II III IV	4 (1.4) 53 (18.7) 226(79.9)
*Precipitating factor of HF (n* = *211)*	
Community-acquired pneumonia Atrial fibrillation Drug discontinuation Acute coronary syndrome Pulmonary tuberculosis Others*	122 (57.8) 60 (28.4) 49 (23.2) 17 (8.0) 17 (8.0) 64 (30.3)

*Exacerbated chronic obstructive pulmonary disease and asthma, severe pulmonary hypertension, left ventricular thrombus, thyroid disorder

### Laboratory and imaging investigations

**Fig. 4:**
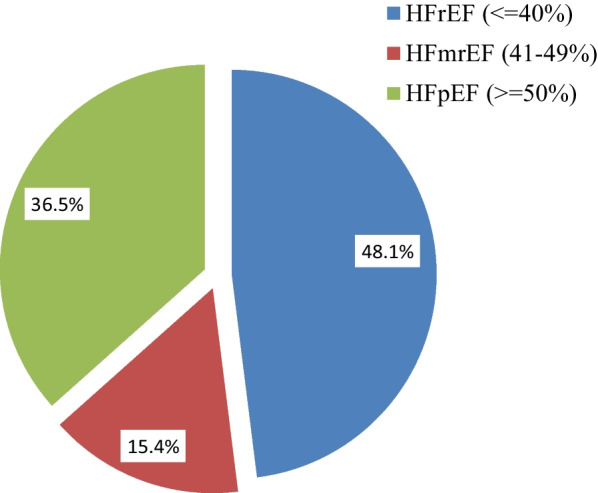
Classification of heart failure based on ejection fraction admitted to JUMC and AURH, Ethiopia between September 01, 2020 and March 01, 2021. HFmrEF-heart failure with mid-range ejection fraction, HFpEF-heart failure with preserved ejection fraction, HFrEF-heart failure with a reduced ejection fraction

Among 283 HF patients assessed in this study, 208 (73.5%) had a recorded echocardiographic finding with the results of 100 (48.1%) HFrEF, 76 (36.5%) HFpEF, and 32 (15.4%) had a mid-range ejection fraction (Fig. 4). Of these, there were 138 (59.2%) abnormal ECG, 69 (27.4%) elevated serum creatinine, and 77 (43.0%) hyponatremia. There was 136 (50.9%) low hemoglobin level record, of these 36 (12.7%) were diagnosed with significant anemia (Table 3).

**Table 3:** Laboratory and imaging investigations values of heart failure patients admitted to JUMC and AURH, Ethiopia between September 01, 2020 and March 01, 2021

Parameters	Frequency (%)
*Electrocardiogram (n* = *233)*	
Normal Abnormal	95 (40.8) 138 (59.2)
Troponin (n = 67) ≥ 1.5 ng/L	28 (41.8)
Serum creatinine (n = 252) > 1.2 mg/Dl	69 (27.4)
*Thyroid-stimulating hormone (µU/mL) (n* = *26)*	
< 0.5 > 4.7	11 (42.4) 1 (3.8)
*Liver function test (IU/L) (n* = *206)*	
AST > 40 ALT > 40	72 (35.0) 57 (27.7)
Sodium (n = 179) < 135 mEq/L	77 (43.0)
*Potassium (mEq/L) (n* = *179)*	
< 3.5 > 5.5	37 (20.7) 11 (6.1)
*Complete blood count (n* = *267)*	
Leukocytosis (WBC > 12,000 cell/mm^3^)	38 (14.2)
Low hemoglobin (mg/dL)*	136 (50.9)

a = Sinus tachycardia, atrial fibrillation, atrial flutter, sinus bradycardia, ST elevation/depression

* < 13 for males and < 12 for females as per world health organization (WHO) criteria [15]

### Management

Of a total of 283 patients enrolled in the current study, 170 (60.1%) had past medication history, 283 (100.0%) had in-hospital current medications records, and 251 (88.7%) had documented discharge medications. During hospitalization, diuretics (98.9%) were used as the predominant class of drugs. It is also used most commonly than other classes of drugs during both pre-hospitalization and post-hospitalization. ACEIs/ARBs 169 (67.3%) and beta-blockers 162 (64.5%) were commonly used during post-hospitalization. During hospitalization, 188 (66.4%) patients were prescribed antibiotics, among these, 145 (77.1%) were diagnosed as significantly infectious (Table 4).

**Table 4:** Management (past, in-hospital, and discharge medications) of heart failure patients admitted to JUMC and AURH, Ethiopia between September 01, 2020 and March 01, 2021

Medications	Frequency (%)		
	Past medication history medications	In-hospital medications	Discharge
	(n = 170)	(n = 283)	(n = 251)
ACEIs/ARBs	99 (58.2)	138 (48.8)	169 (67.3)
Diuretics	147 (86.5)	280 (98.9)	240 (95.6)
Beta-blockers*	77 (45.3)	132 (46.6)	162 (64.5)
CCBs	15 (8.8)	30 (10.6)	30 (12.0)
MRAs	21 (12.4)	46 (16.3)	27 (10.8)
Cardiac glycoside	16 (9.4)	32 (11.3)	31 (12.4)
Statins	57 (33.5)	126 (44.5)	120 (47.8)
Anticoagulant	18 (10.6)	71 (25.1)	4 (1.6)
Antiplatelet	47 (27.6)	125 (44.2)	98 (44.6)
Antibiotics	-	188 (66.4)	-

ACEIs/ARBs-angiotensin converting enzyme inhibitors or angiotensin receptor blockers, CCBs-calcium channel blockers, MRAs-mineralocorticoid receptor antagonists

*Metoprolol and carvedilol

### Outcomes

#### In-hospital events

Arrhythmia 69 (24.4%) was the most common cause of in-hospital complications of HF, followed by acute kidney injury 38 (13.4%) and cardiogenic shock 20 (7.1%). The median length of hospital stay was 14 (IQR = 9, 19) days with 25 (8.8%) in-hospital deaths (Table 5).

**Table 5:** In-hospital events (complications, length of stay, mortality) of heart failure patients admitted to JUMC and AURH, Ethiopia between September 01, 2020 and March 01, 2021

Events	Frequency (%)
*In-hospital complication HF (n = 135)*	
Arrhythmia Acute kidney injury Cardiogenic shock Acute ischemia Hospital-acquired infection	69 (24.4) 38 (13.4) 20 (7.1) 17 (6.0) 7 (2.5)
In-hospital mortality	25 (8.8)
Hospital stay, median (interquartile range)	14 (IQR = 9, 19) days

### Predictors of 90-day all-cause mortality

**Fig. 5:**
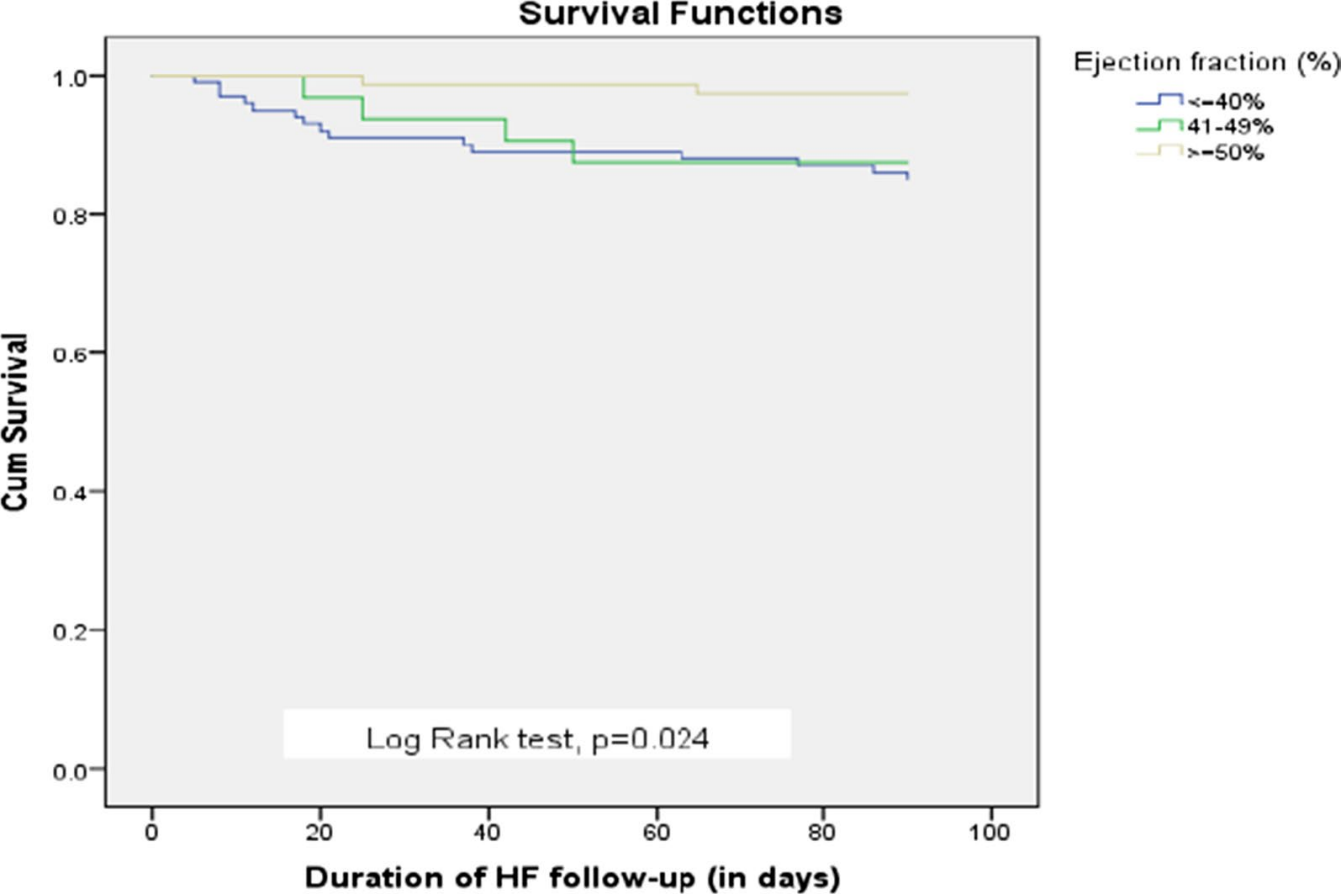
Kaplan Meier survival curves at 90-day all-cause mortality for patients with preserved, mid-range, and reduced ejection fraction of heart failure patients admitted to JUMC and AURH, Ethiopia between September 01, 2020, to March 01, 2021

The Kaplan Meier survival curves (Fig. 5) show there was a significant difference in the survival status of patients with HFrEF, HF with mid-range EF, and HFpEF (Log Rank test, P = 0.024). The variables that showed significant association (P value < 0.25) on univariate Cox regression analysis were alcohol consumption, atrial fibrillation, cardiogenic shock, anemia, and reduced ejection fraction which were significantly associated with 90-day all-cause mortality. On other hand, the variables that were identified by multivariate Cox regression analysis were the presence of hypertension, alcohol drinking, cardiogenic shock, reduced ejection fraction, and absence of ACEIs/ARBs, which significantly predicted 90-day all-cause mortality (Table 6).

**Table 6:** A 90-day all-cause mortality independent predictor of heart failure patients admitted to JUMC and AURH, Ethiopia between September 01, 2020 and March 01, 2021

Univariate analysis				Multivariate analysis	
Variables	Frequency (%)	HR (95% CI)	P value	HR (95% CI)	P value
Alcohol drinking	19(28.4)	1.7 (0.8–3.7)	0.167	2.8 (1.1–7.8)	**0.043**
Hypertension	16(15)	1.6 (0.7–3.2)	0.243	3.7 (1.2–11.6)	**0.025**
CRVHD	8(16)	1.8 (0.7–4.3)	0.223	1.9 (0.5–7.4)	0.387
Atrial fibrillation	8(13.3)	4.3 (1.5–12.5)	**0.007**	2.1 (0.64–7.0)	0.218
Anemia	6(16.7)	1.9 (0.8–4.6)	0.167	2.7 (0.8–9.9)	0.130
CAP	16(13.1)	1.7 (0.8–3.5)	0.173	1.5 (0.5–4.5)	0.512
Cardiogenic shock	10(50)	8.8(4.1–18.9)	** < 0.001**	8.7 (3.2–20.8)	** < 0.001**
Rehospitalization	10(15.4)	1.7 (0.8–3.7)	0.159	1.3 (0.4–3.7)	0.684
HFrEF ≤ 40%)	15(15)	1.0 (1.0–1.1)	0.196	1.5 (1.1–3.8)	**0.014**
ACEIs/ARBs	29(25.4)	0.6 (0.3–1.2)	0.122	0.02 (0.0–0.2)	** < 0.001**

*P*-value < 0.05 considered as statically significant and shown in bold, means they have strong association with HF mortality

ACEIs/ARBs-angiotensin converting enzyme inhibitors/angiotensin receptor blockers, CAP-community acquired pneumonia, CI-confidence interval, CRVHD-chronic valvular heart disease, HR-hazard ratio

## Discussion

The 90-day all-cause mortality in HF patients hospitalized in two Ethiopian tertiary hospitals was observed in this study. Ischemic heart disease (47%) and hypertension (51.9%) were the most frequent underlying and co-morbid diseases, respectively. Among HF patients assessed in the current study, 57.8% were precipitated by pneumonia. The 90-day all-cause mortality of this study was 10.2%.

Among 283 patients assessed, 66.8% were younger than 65 years old, with a mean (± SD) age of 52.4 ± 17.9 years. When we compare the demographic characteristics of patients with heart failure revealed by this study to data from studies conducted in the USA, almost 75% of HF patients were > 65 years, with a mean (± SD) age of 69.1 ± 13.5 years, and Spain, 66.7% of HF patients were > 65 years, with a mean (± SD) age of 72.8 ± 11.2 years [16, 17]. Living styles, socioeconomic status of patients, and environmental, and genetic factors were believed to be the possible causes of these disparities. A study conducted in 11 Asian regions showed that more than 50% of HF patients were < 65 years old [18]. On the other hand, comparable findings reported in Ethiopia showed that more than 66% of participants were < 65 years old [19, 20].

Approximately 80% of the HF patients in this study had NYHA class IV. A study conducted in Spain and Japan showed that 75% and 36.1% of patients had NYHA class III–IV respectively, which is relatively different from the current study [21, 22]. On the other hand, a study conducted in Ethiopia showed that 90.3% of NYHA class IV patients, which is relatively consistent with the current study [23]. This demonstrates that HF patients in Ethiopia come with later-stage symptoms when they visit a health facility. The socio-economic status of the patients, distance from the hospital, low level of awareness about the disease, and the quality of health care services might be all likely factors in these late-stage presentations.

Hypertension was shown to be the most common risk factor for HF in this study (51.9%). According to a study conducted in the USA (75.6%), New Zealand and Singapore (67.8%), Tanzania (41%), and South Africa (46%), hypertension was the most common risk factor for HF patients [16, 24–26]. In this study, the possible causes of the high proportion of hypertension as a risk factor for HF were a lack of adherence to salt intake recommendations, inadequate quality of health care services, the poor lifestyle of the patients, poor diet monitoring habits, race variation, environmental, and genetic factors. On the other hand, when compared to the findings of a previous study conducted in Ethiopia by different scholars, the most common risk factor for heart failure was valvular heart disease, followed by hypertensive heart disease, coronary heart disease, and ischemic heart disease, which is not in line with the current study [19, 27].

The most common precipitating variable in the current study was pneumonia (57.8%), followed by atrial fibrillation, drug discontinuation, and acute coronary syndrome. A study conducted in Ethiopia revealed that pneumonia (42%) was the most common cause of HF exacerbation, followed by arrhythmia, anemia, myocardial infarction, and drug discontinuation [28]. Lack of adult pneumococcal and influenza vaccination for patients with chronic disease and home surrounding hygiene of the patients might be the likely causes of HF exacerbation by pneumonia.

In the current study, diuretics were the most commonly prescribed drug for HF patients, followed by ACEIs/ARBs, beta-blockers, statins, antiplatelet, mineralocorticoid receptor antagonists, digoxin, and calcium channel blockers. A similar study in Ethiopia showed that diuretics were used most commonly, followed by beta-blockers, ACEIs/ARBs, mineralocorticoid receptor antagonists, digoxin, and statins [19]. Another study from Japan, New Zealand and Singapore showed that diuretics were used most commonly, followed by beta-blockers, ACEIs/ARBs, mineralocorticoid receptor antagonists, and statins for HF patients [22, 24]. On the other hand, findings from studies in the USA and Spain revealed that HF patients were treated with ACEIs/ARBs most commonly, followed by beta-blockers, diuretics, digoxin, and statins, which is relatively not consistent with our study [16, 20]. The increased consumption of diuretics in Ethiopia was possible due to the presentation of HF patients with congestion as a result of non-adherence to salt intake recommendations and the drugs' easy availability.

This finding revealed a 90-day all-cause mortality rate of 10.2%, which is relatively similar to a study conducted in the USA's 11.5% of all-cause mortality rate [29]. The slight discrepancies in these studies were due to the number of participants enrolled, ethnic variation, and genetic factors. Another study that is not in line with the current study was reported in Ethiopia with 17.2% in-hospital mortality of 4 months prospective observational study [20] and 17% report from Japan with 19 months of follow-up [22]. Specifically, all-cause mortality of the present study was lower than study findings from Japan, which might be the most likely cause of differences in follow-up period length, study design, the number of study participants enrolled, and environmental, and genetic factors.

In the current study, multivariate Cox regression analysis showed that the independent predictors of 90-day all-cause mortality in HF patients were alcohol drinking, presence of hypertension, reduced ejection fraction, cardiogenic shock, and absence of ACEIs/ARB's medications. In a comparable study report from the international registry (REPORT-HF) across all regions, the common predictors of worse 1-year mortality were lower systolic blood pressure and not receiving ACEIs or β blockers at discharge [30]. A similar study done in Israel exhibited that the predictors of all-cause mortality were admission systolic blood pressure ≥ 140 mmHg [31]. Another study conducted in Zambia showed that the predictors of all-cause mortality were left ventricular ejection fraction < 40% [32].

### Strengths and limitations of the study

The lack of laboratory value on brain natriuretic peptide, which is sensitive to HF diagnosis, was one of the study's limitations. Furthermore, heart investigations and laboratory results such as an echocardiogram, electrocardiogram, cardiac troponin, serum electrolyte, and liver function tests were not obtained fully. The difference in reference range among different laboratory settings that results from facilities' machine calibration and genetic factors was another limitation of this study. The cause of death after discharge could not be determined in detail. Despite these limitations, our study provides vital information on the clinical characteristics, management, and prognosis of HF patients.

## Conclusion

High 90-day all-cause mortality was observed among heart failure patients in the present study. The most common co-morbid diseases and etiology in patients admitted with HF were hypertension and ischemic heart disease. Furthermore, pneumonia was the principal precipitating factor of HF. In the current study, the majority of HF patients were treated with diuretics. Alcohol drinking, presence of hypertension, reduced ejection fraction, cardiogenic shock, and the absence of ACEIs/ARBs medications were all predictors of poor treatment outcomes for whom closer monitoring or restriction of alcohol consumption, early management of hypertension, HFrEF, cardiogenic shock, and providing ACEIs/ARBs drugs for all HF patients would be recommended to improve these poor treatment outcomes.

## Data Availability

The data set that was used to support the finding of this study will be made available from the corresponding author upon reasonable request.

## Abbreviations

AURH: Ambo University Referral Hospital
ACEI: Angiotensin converting enzyme inhibitors
ARB: Angiotensin receptor blockers
CVD: Cardiovascular diseases
JUMC: Jimma University Medical Center
HF: Heart failure
HFpEF: Heart failure with a preserved ejection fraction
HFrEF: Heart failure with a reduced ejection fraction
